# Analysis of Time Utilization in the Operating Theater and Factors Contributing to Delays in Scheduled Surgeries

**DOI:** 10.7759/cureus.83671

**Published:** 2025-05-07

**Authors:** Ghina Awais, Lajpat Rai, Ali Naqi, Ihsan Ahmed, Shahzad Asif, Aiman Aamir, Sidra Gulzar, Nazia Lodhi, Ghina Shamsi, Sheeraz S Siddiqui

**Affiliations:** 1 Department of General Surgery, Indus Hospital & Health Network, Karachi, PAK; 2 Department of General Surgery, Wrexham Maelor Hospital, Wrexham, GBR; 3 Department of Hepatobiliary and Liver Transplant, Shifa International Hospital Islamabad, Islamabad, PAK; 4 Department of Breast Surgery, The Indus Hospital, Karachi, PAK

**Keywords:** associated factors, operation theater, operation theater delay, optimal utilization, raw utilization time

## Abstract

Introduction: The operation theater (OT) is a significant expenditure in hospital budgets, necessitating maximum utilization for optimal cost efficiency. This study aims to determine the percentage of raw utilization time of general surgical theaters at Indus Hospital, Karachi.

Objective: The objective of this study is to assess the raw utilization percentage of the general surgery OT.

Materials and methods: The cross-sectional study was conducted between February 20, 2022, and August 25, 2022 at the Department of General Surgery, the Indus Hospital, Karachi. All elective surgical procedures performed between 08:00 AM and 05:00 PM that met the inclusion criteria were analyzed. Raw utilization time was calculated from the collected data.

Results: The majority of patients were female (62.7%). The raw utilization time was 83.8%. The most common reasons for discrepancies between actual and projected procedure times were technical difficulties (26.6%), equipment-related issues (16.7%), and staff shortages (2.6%).

Conclusion: This study identifies avoidable factors contributing to discrepancies in procedure timing. Improved communication among surgeons, anesthesiologists, and nursing staff prior to surgeries could help mitigate delays.

## Introduction

The operating theater (OT) complex is a vital component of hospital operations, accounting for a substantial portion of healthcare expenditures. Approximately 50%-60% of hospital revenue is derived from surgical procedures, underscoring the importance of efficient OT utilization to optimize the cost-benefit ratio [1]. Maximizing OT efficiency requires careful coordination of activities and personnel, as well as minimizing disruptions, particularly last-minute cancellations, which lead to wasted resources and increased stress for patients and healthcare providers [1-3].

An OT serves as a specialized facility where invasive procedures are performed under strict aseptic conditions by trained personnel. Effective management is crucial for ensuring patient safety, comfort, and cost-efficiency [4-6]. Utilization, defined by Donham et al. as the proportion of hours allocated to surgeries during elective hours compared to total available elective hours, is a key measure of OT efficiency [7].

Several studies have reported varying OT utilization rates. Bhaskar et al. found a rate of 72.51% at a tertiary care hospital, with most hours dedicated to elective surgeries and emergency procedures filling unscheduled gaps [8]. Stavrou et al. noted variability in time spent across surgical phases based on procedure complexity [9], while Vinukondaiah et al. reported a utilization rate of 91.5%, identifying cancellations primarily due to time constraints and emergency cases [1]. Additionally, Garg et al. found that approximately 30.3% of scheduled surgeries were canceled on the day of surgery, citing factors such as insufficient theater time and administrative issues [10].

These inefficiencies highlight the need for healthcare administrators to meticulously plan and manage OT resources. Given the high costs associated with operating rooms, including specialized personnel, the focus must be on minimizing idle time and optimizing scheduling [11-13]. Effective OT management necessitates strategic, tactical, and operational planning, distributing OT time across specialties and allocating time blocks within a master surgery schedule [14-16].

The complexity of OT scheduling arises from various constraints, including case mix variability and competing priorities among stakeholders [17]. Most research has focused on elective surgeries, with less emphasis on non-elective cases that can disrupt scheduling [18]. While mathematical modeling has been employed to address surgical planning, practical implementation often falls short [19]. This manuscript aims to explore pathways to improve OT efficiency through standardized processes, enhanced resource planning, and refined scheduling methods [20-25].

## Materials and methods

This cross-sectional study was conducted at the Department of General Surgery, The Indus Hospital, Karachi. The study was carried out at a single center from February 20, 2022, to August 25, 2022. The sample size was determined using OpenEpi version 3.01, with a 5% margin of error, a 95% confidence level. The minimum required sample size was 232 elective surgeries. A consecutive sampling method was employed.

The study included all elective general surgical operations scheduled from 8:00 AM to 5:00 PM (540 minutes). The procedures where anesthesia induction commenced by 4:00 PM were considered. All surgeries experiencing unexpected delays were included, with descriptive analysis performed to understand the reasons for delays. The cases where anesthesia induction occurred after 4:00 PM, emergency surgeries, surgeries with unexpected cancellation, and those performed in theatres other than the specified one were all excluded. 

Following approval from the hospital’s ethical board, data collection commenced. All procedures conducted within the available resource hours were included. Before each procedure, the chief surgeon documented the projected time for the operation. The time periods from pre-anesthesia to recovery were recorded in minutes using a stopwatch by the principal investigator and documented in a questionnaire. Any discrepancies between projected and actual procedure times were noted, along with the factors contributing to these discrepancies.

Data analysis

Data was entered and analyzed using IBM SPSS Statistics for Windows, Version 26 (Released 2019; IBM Corp., Armonk, New York, United States). The normality of continuous variables (e.g., raw utilization time, room turnover time, time spent on the actual procedure, time spent on supportive services, total projected time, and actual time) was assessed using the Shapiro-Wilk test. Normally distributed variables were presented as mean and standard deviation (SD), while non-normally distributed variables were reported as median and interquartile range (IQR). Frequencies and percentages were calculated for qualitative variables such as designation, gender, and reasons for discrepancies between projected and actual times. Stratification was applied to control for effect modifiers such as gender, designation, room turnover time, time spent on the actual procedure, time spent on supportive services, total projected time, and actual time. After stratification, the Mann-Whitney U test was employed, with a significance level set at p < 0.05.

## Results

A total of 233 cases were scheduled during the study period. The majority of patients included in the study were female (62.7%). The total resource time amounted to 29,000 minutes, with a mean "raw utilization" time of 104.73 ± 66.43 minutes. The overall raw utilization time for the 233 cases was 24,304 minutes, resulting in a raw utilization percentage of 83.80%. The mean time spent on supportive services was 38.37 ± 27.04 minutes, contributing to a total of 8,940 minutes. The mean procedure time was 64.97 ± 53.14 minutes, amounting to a total procedure time of 15,139 minutes. The mean room turnover time was 50.89 ± 33.33 minutes, resulting in a total of 11,705 minutes. Detailed statistics are presented in Table 1.

**Table 1:** Time utilization and procedure analysis

Variable	Mean (mins)	SD (mins)	Median (mins)	IQR (mins)	Min (mins)	Max (mins)
Raw Utilization Time	104.73	66.43	91.50	66-130	10	540
Actual Time of Procedure	66.62	55.22	58.00	31-81	02	470
Room Turnover Time	50.89	33.33	41.50	30-60	10	230
Time Spent on Procedure	64.97	53.14	58.00	32-61	02	478
Time Spent on Supportive Services	38.37	27.04	35.00	20-49	03	234
Projected Time	61.97	41.99	60.00	40-75	05	420
Raw Utilization Time	104.73	66.43	91.50	66-130	10	540

The analysis of discrepancies between actual and projected procedure times revealed the most common reasons as technical difficulty (26.6%), equipment-related issues (16.7%), and staff shortages (2.6%). Notably, 53.6% of discrepancies were due to unavailable resources or other unspecified reasons (Table 2).

**Table 2:** Distribution of reasons for discrepancy

Reason of Discrepancy	N (%)
Equipment Related	39 (16.7)
Technical Difficulty	62 (26.6)
Staff Shortage	6 (2.6)
Not Available	125 (53.6)
Others	(0.4)

In terms of raw utilization time, gender differences were observed. Male patients had a significantly higher median raw utilization time of 110 minutes (IQR: 75-140) compared to female patients, whose median was 90 minutes (IQR: 55-124). The difference was statistically significant (Mann-Whitney U = 5252.500, p = 0.027) (Table 3).

**Table 3:** Stratification of time variables by group and category

Comparison Group	Category	Median (IQR)	Mann-Whitney U	Wilcoxon W	Z-value	p-value
Gender	Male	110 (75-140)	5252.500	15983.500	-2.208	0.027
	Female	90 (55-124)				
Designation	Consultant	98 (73-131)	5591.500	16322.500	-1.527	0.127
	Resident	90 (57-128)				
Room Turnover Time	≤55 mins	90 (54-124)	3807.000	18513.000	-3.287	0.004
	>55 mins	113 (80-142)				
Actual Time of Procedure	≤55 mins	65 (45-80)	652.000	7207.000	-11.924	<0.001
	>55 mins	125 (108-154)				
Time Spent on Procedure	≤55 mins	65 (45-80)	656.500	7097.500	-11.912	<0.001
	>55 mins	124 (107-153)				
Time Spent on Supportive Service	≤55 mins	85 (53-115)	933.000	19269.000	-7.785	<0.001
	>55 mins	154 (124-209)				
Projected Time	≤55 mins	50 (39-74)	1022.500	4508.500	-10.562	<0.001

For room turnover time, patients with turnover times greater than 55 minutes showed a significantly higher median raw utilization time (113 minutes, IQR: 80-142) compared to those with turnover times of 55 minutes or less (median 90 minutes, IQR: 54-124), with a Mann-Whitney U of 3807.000 (p = 0.004).

Similarly, for actual time of procedure and time spent on procedure, those with procedure times longer than 55 minutes had a significantly higher median raw utilization time (125 minutes, IQR: 108-154 for procedure time; 124 minutes, IQR: 107-153 for time spent on procedure), compared to those with times of 55 minutes or less, with both p-values < 0.001.

The time spent on supportive services was also significantly greater for patients whose procedure times exceeded 55 minutes, with a median of 154 minutes (IQR: 124-209), compared to those with procedure times of 55 minutes or less (median: 85 minutes, IQR: 53-115), with p < 0.001.

Lastly, when comparing projected time, those with procedure times longer than 55 minutes had a higher median projected time of 117 minutes (IQR: 90-150) versus those with 55 minutes or less (median: 50 minutes, IQR: 39-74), with a statistically significant p-value of < 0.001.

These findings underscore the significant impact of various factors, such as gender, room turnover time, actual procedure time, time spent on the procedure, and supportive services, on the raw utilization time. Further stratified analysis is detailed in Table 3.

## Discussion

Our study demonstrated an overall raw utilization rate of 83.80%, indicating substantial efficiency while highlighting room for improvement in maximizing operating time. Limited operating hours, particularly with no elective surgeries scheduled on Sundays and public holidays, necessitate careful analysis and management of OT resources to meet the diverse needs of patients, surgeons, and staff [26].

The significance of structured OT management is well-documented, with roots tracing back to the late 1970s [27]. An effectively functioning OT requires substantial resources, including equipment, personnel, and operational protocols [28]. Our findings resonate with previous studies, such as those by Vinukondaiah et al., who reported high utilization rates of 91.5%. Although our percentage was slightly lower, it underscores a consistent trend of effective resource management across institutions [1].

In analyzing time utilization, we found that the mean procedure time was 64.97 minutes, while the mean room turnover time was 50.89 minutes. This aligns with studies by Jan et al. and Haiart et al., which highlighted similar distributions of time spent on surgery and turnover activities, revealing a common challenge in maintaining efficiency during transitions between cases [26,27].

Delays in starting procedures significantly impact overall OT utilization. Our study identified technical difficulties (26.6%) and equipment-related issues (16.7%) as the primary reasons for discrepancies between projected and actual times. This is consistent with findings from Vinukondaiah et al., where 43.6% of surgical lists started later than scheduled, predominantly due to delays in patient transfers to the OT [1]. These insights suggest a pressing need for improved communication between nursing staff and surgeons regarding pre-operative instructions and patient readiness [26].

Staffing impacts on OT efficiency cannot be overlooked. Understaffing has been identified as a critical barrier to optimal utilization, as reported by Haiart et al. [27]. Our data reinforce this notion, suggesting that a more robust staffing model could mitigate delays and enhance resource management. Introducing an OT manager to oversee scheduling and operational flow may further streamline processes, reduce cancellations, and improve overall efficiency [27].

Interestingly, our study found no cancellations due to patient-related issues, contrasting with literature indicating that non-clinical reasons often lead to significant cancellation rates [29]. This discrepancy may reflect the effectiveness of our scheduling practices or the specific context of the healthcare facility.

The variability of surgical case durations poses additional challenges to predicting actual utilization. Even routine procedures can present unpredictability in timing, complicating scheduling efforts [27]. Continuous audits of OT utilization are essential to identify trends, inform strategic adjustments, and enhance resource allocation. By focusing on improved communication among surgical teams and better scheduling practices, we can aspire to elevate OT efficiency and ultimately enhance patient care.

## Conclusions

This study demonstrates a promising raw utilization rate in the general surgery OT at the Indus Hospital. However, identified areas for improvement, such as technical difficulties and equipment-related issues, contribute to delays. These findings underscore the importance of enhancing operational efficiency in the OT to maximize resource utilization and improve patient care. Future research should aim to address the underlying causes of delays and explore innovative solutions to optimize surgical scheduling and patient flow.
